# Diagnosis of Monkeypox Disease Using Transfer Learning and Binary Advanced Dipper Throated Optimization Algorithm

**DOI:** 10.3390/biomimetics8030313

**Published:** 2023-07-16

**Authors:** Amal H. Alharbi, S. K. Towfek, Abdelaziz A. Abdelhamid, Abdelhameed Ibrahim, Marwa M. Eid, Doaa Sami Khafaga, Nima Khodadadi, Laith Abualigah, Mohamed Saber

**Affiliations:** 1 Department of Computer Sciences, College of Computer and Information Sciences, Princess Nourah bint Abdulrahman University, P.O. Box 84428, Riyadh 11671, Saudi Arabia; 2 Computer Science and Intelligent Systems Research Center, Blacksburg, VA 24060, USA; 3 Department of Communications and Electronics, Delta Higher Institute of Engineering and Technology, Mansoura 35111, Egypt; 4 Department of Computer Science, College of Computing and Information Technology, Shaqra University, Shaqra 11961, Saudi Arabia; 5 Department of Computer Science, Faculty of Computer and Information Sciences, Ain Shams University, Cairo 11566, Egypt; 6 Computer Engineering and Control Systems Department, Faculty of Engineering, Mansoura University, Mansoura 35516, Egypt; 7 Faculty of Artificial Intelligence, Delta University for Science and Technology, Mansoura P.O. Box 11152, Egypt; 8 Department of Civil and Architectural Engineering, University of Miami, Coral Gables, FL 33146, USA; 9 Computer Science Department, Prince Hussein Bin Abdullah Faculty for Information Technology, Al al-Bayt University, Mafraq 25113, Jordan; 10 Department of Electrical and Computer Engineering, Lebanese American University, Byblos 13-5053, Lebanon; 11 Hourani Center for Applied Scientific Research, Al-Ahliyya Amman University, Amman 19328, Jordan; 12 MEU Research Unit, Middle East University, Amman 11831, Jordan; 13 Applied Science Research Center, Applied Science Private University, Amman 11931, Jordan; 14 School of Computer Sciences, Universiti Sains Malaysia, Gelugor 11800, Malaysia; 15 School of Engineering and Technology, Sunway University Malaysia, Petaling Jaya 27500, Malaysia; 16 Electronics and Communications Engineering Department, Faculty of Engineering, Delta University for Science and Technology, Mansoura P.O. Box 11152, Egypt

**Keywords:** biological mechanism, monkeypox detection, deep learning, transfer learning, feature selection, dipper throated optimization

## Abstract

The virus that causes monkeypox has been observed in Africa for several years, and it has been linked to the development of skin lesions. Public panic and anxiety have resulted from the deadly repercussions of virus infections following the COVID-19 pandemic. Rapid detection approaches are crucial since COVID-19 has reached a pandemic level. This study’s overarching goal is to use metaheuristic optimization to boost the performance of feature selection and classification methods to identify skin lesions as indicators of monkeypox in the event of a pandemic. Deep learning and transfer learning approaches are used to extract the necessary features. The GoogLeNet network is the deep learning framework used for feature extraction. In addition, a binary implementation of the dipper throated optimization (DTO) algorithm is used for feature selection. The decision tree classifier is then used to label the selected set of features. The decision tree classifier is optimized using the continuous version of the DTO algorithm to improve the classification accuracy. Various evaluation methods are used to compare and contrast the proposed approach and the other competing methods using the following metrics: accuracy, sensitivity, specificity, *p*-Value, N-Value, and F1-score. Through feature selection and a decision tree classifier, the following results are achieved using the proposed approach; F1-score of 0.92, sensitivity of 0.95, specificity of 0.61, *p*-Value of 0.89, and N-Value of 0.79. The overall accuracy of the proposed methodology after optimizing the parameters of the decision tree classifier is 94.35%. Furthermore, the analysis of variation (ANOVA) and Wilcoxon signed rank test have been applied to the results to investigate the statistical distinction between the proposed methodology and the alternatives. This comparison verified the uniqueness and importance of the proposed approach to Monkeypox case detection.

## 1. Introduction

Early detection is essential to treat and prevent the spread of monkeypox effectively. Deep learning is a kind of machine learning that employs convolutional neural networks to study and learn from large datasets. The diagnosis of infections like monkeypox has been greatly aided by this method. Deep learning algorithms can sift through mountains of data from medical imaging, laboratory testing, and patient records to better diagnose disease. The interactions between the monkeypox virus and the host cells are fundamental to the disease’s complicated molecular mechanisms. A virus infects a host cell and then replicates inside of it, causing the host cell to die and the virus to spread to neighboring cells and tissues. When the immune system detects a virus or infected cells, it responds by making antibodies and immune cells to destroy them. Hopefully, by delving into the molecular workings of monkeypox, more effective therapies and vaccinations might be created. Deep learning can be applied to massive amounts of molecular and genetic data to find promising areas to focus on when designing treatments or vaccines. Researchers can benefit from deep learning’s ability to zero in on specific disease-related chemicals and pathways to create more precise and tailored treatments. It is crucial to understand the basic underpinnings of monkeypox to understand how deep learning can detect the disease. Effective therapies and vaccinations for monkeypox require an understanding of the disease’s basic mechanisms, which the use of deep learning algorithms for detection and diagnosis can aid. These strategies can potentially enhance the control of monkeypox and other viral infections [1,2,3].

The monkeypox virus, responsible for this illness, is a member of the Poxviridae family and the orthopoxviral genus. Similarly, smallpox is caused by the Variola virus, a Poxviridae family member. The cowpox virus causes bovine smallpox, and vaccines are made with the Vaccinia virus. While it is commonly referred to as “monkeypox”, the virus that causes it actually originated in rodents. The virus known as monkeypox was first identified in 1958 after two outbreaks with symptoms identical to those of smallpox occurred in monkey colonies housed for scientific purposes. In 1970, the monkeypox virus was recognized for the first time in a human host. Since those times, monkeypox incidence has been extremely low throughout Africa. Transmission of monkeypox, which has been observed for a considerable amount of time in West and Central Africa due to the region’s high concentration of tropical rainforests, has rarely occurred due to animals shipped from the region. The disease has recently spread and been identified in a wider variety of people and geographic locations than ever before. Because of the terrible impacts of the COVID-19 pandemic, monkeypox cases have begun to be closely monitored, even though they are not yet at the pandemic stage, and indicate an epidemic spread [4,5,6,7].

Monkeypox is characterized by a rash lasting anywhere from one to five days. Rash symptoms frequently manifest themselves initially on the face before spreading elsewhere. Lesions in the vaginal area, eyes, and intraoral mucosa have been reported in certain patients. The rashes of this illness often seem like those of chickenpox, leading to misdiagnoses. These rashes start as water-filled blisters but eventually heal into crusty areas. Lesions can manifest in a wide variety of ways; for some people, it is a widespread rash of small blisters, while for others it is more extensive. Lesions may join together to form widespread rashes on the skin’s surface in severe situations. Depending on the severity of the sickness, the rashes typically vanish entirely in two to four weeks, and the disease heals.

Convolutional neural networks (CNNs) are widely employed in academic studies in deep learning, as is evident when examining the most cutting-edge technologies in image processing and classification. When gathering insights, images are a common input for CNN, a type of deep learning model. It records the results of several processes on the image to categorize the future inferences that might be drawn from it. In 1988, the authors of [8] presented the first CNN structure using the LeNet design, which was refined until 1998. CNN algorithms find use in various areas, including NLP (natural language processing) and biomedicine (particularly in processing images and sounds). The best outcomes have been achieved, particularly in image processing. Using CNN, the authors of a recent paper [9,10,11] were able to bring the error rate on the MNIST dataset down to 0.23%.

Early treatment is most effective when skin lesions caused by the monkeypox disease are quickly identified and distinguished from other diseases characterized by similar lesions. To reduce the spread of monkeypox, mobile devices should be able to tell the lesions of that disease apart from those of other conditions that cause similar skin lesions. This research aims to accurately categorize monkeypox impact on skin lesions, which have begun to spread fast worldwide, by identifying them rapidly and accurately using transfer learning techniques. End users can determine whether or not they have monkeypox by taking an image with their phone and running it through the transfer-learning-trained TFLite model.

The structure of this paper is organized as follows. Section 2 discusses the previous research efforts on monkeypox. In Section 3, the proposed model is illustrated and explained. The experimental results and performance evaluation are presented in Section 4 as comparisons to various benchmark schemes. Section 5 analyzes the study by contrasting it with other research. Section 6 wraps up the discussion and future perspectives.

## 2. Literature Review

Previous research was reviewed to use deep learning to categorize monkeypox. Researchers generated skin lesion images from open-source websites to boost the amount of data for use in deep learning to classify monkeypox skin lesions. They used data augmentation techniques, including k-fold cross-validation. Pre-trained versions of the VGG-16, ResNet50, and InceptionV3 models were tested to categorize monkeypox and other diseases. The highest rate of accuracy was found in ResNet50; hence, that model was utilized. A 0.82 F1 Score was calculated for the ResNet50. The Grad-Cam and LIME methods have been implemented into the Xception transfer learning model [12,13,14]. The Xception and DenseNet models have developed a community-based strategy; it is based on cooperation. The investigation was conducted using the proposed ensemble approach, with average precision, recall, F1 score, and accuracy of 85.44%, 85.47%, and 87.13%, respectively, based on the performance scores acquired from trials on a publicly available dataset.

To improve the accuracy of monkeypox detection, scientists [15] standardized the transfer learning approach. Using GitHub, they distributed their image database to the public. The data used to build the dataset were culled from various sources on the web. After utilizing the VGG16 model to achieve the area under the curve (AUC) values between 0.88 and 0.97, they presented a modified model version incorporating data from two studies. Another study focused on human–monkey disease classification from skin lesion images using pre-trained deep mesh on mobile application [16,17,18]. It is possible to classify some image data using the help of an Android app for mobile devices and some transfer learning techniques. No improvements were made to the authors’ original version of the MobileNetv2 model. A dataset was accessed through a public Kaggle competition. Researchers utilized models such as ResNet18, GoogleNet, EfficientNetb0, NasnetMobile, ShuffleNet, and MobileNetv2. An accuracy of 0.91 and an F1-score of 0.90 were attained on the MobileNetv2 model.

Classification studies of skin lesion images significantly benefit from combining convolutional neural networks (CNNs) with transfer learning approaches. Convolutional neural networks trained using transfer learning were investigated in another study [19,20,21] to detect Lyme disease from images of skin lesions; the results showed an AUC of 0.91, a sensitivity of 0.83, an accuracy of 0.87, and a specificity of 0.80. Automatic detection of erythema migrans and other skin lesions using deep learning algorithms in detecting Lyme disease was studied in another study by [22,23] within the context of skin lesion classification. Hence, with deep learning, an accuracy of 0.86 and an AUC score of 0.95 were attained. This research aimed to use CNN and transfer learning techniques to accurately, sensitively, and selectively identify monkeypox lesions as distinct from other comparable skin disease lesions [24,25,26].

Increased classification precision is one potential outcome of applying ML methods. The two algorithms were evaluated on a publicly available monkeypox dataset, where they were found to classify monkeypox with an average accuracy of 98.8 percent, outperforming the best-competing algorithms. Medical imaging has recently benefited from applying DL methods to improve disease diagnostic accuracy. Many studies have employed CNNs to accurately categorize skin lesions as benign or malignant. To classify skin cancer, for instance, in [27,28,29,30], a CNN was trained using a dataset of images of skin lesions, and an accuracy level of 96.3% was reached. While deep learning (DL) is promising for monkeypox image analysis detection and classification, knowledge gaps must still be filled. To begin, comprehensive data sets are scarce. Past research has often relied on sample sizes that are too small to portray the whole spectrum of monkeypox variation accurately. Because of this, it is possible that overfitting took place, resulting in less accurate results overall [31,32,33]. In addition, much of the earlier research did not employ a validation set to ensure the models were not overfitted to the training data, which could reduce their accuracy when applied to new data. Some categorization methods have restrictions that make their usage undesirable. However, other classification methods, including support vector machines (SVM), naive Bayes, decision tree (DT), k-nearest neighbors (KNN), and random forest, may also be useful for monkeypox classification, even though CNNs have been utilized exclusively in prior studies. Fewer still are real-world data applications that can make use of this information. To the best of our knowledge, however, there has been no previous work on employing DL to detect monkeypox by image analysis. Authors in [34] suggested a deep learning strategy for detecting monkeypox from skin lesion images, which stands out among the rest of the literature. But they could only utilize it on a tiny dataset and did not employ transfer learning. This research seeks to close a knowledge gap by creating a DL algorithm for categorizing monkeypox through image analysis [35,36,37,38,39,40]. Table 1 presents a review of the relevant works related to the problem of monkeypox diagnosis.

## 3. The Proposed Methodology

**Figure 1:**
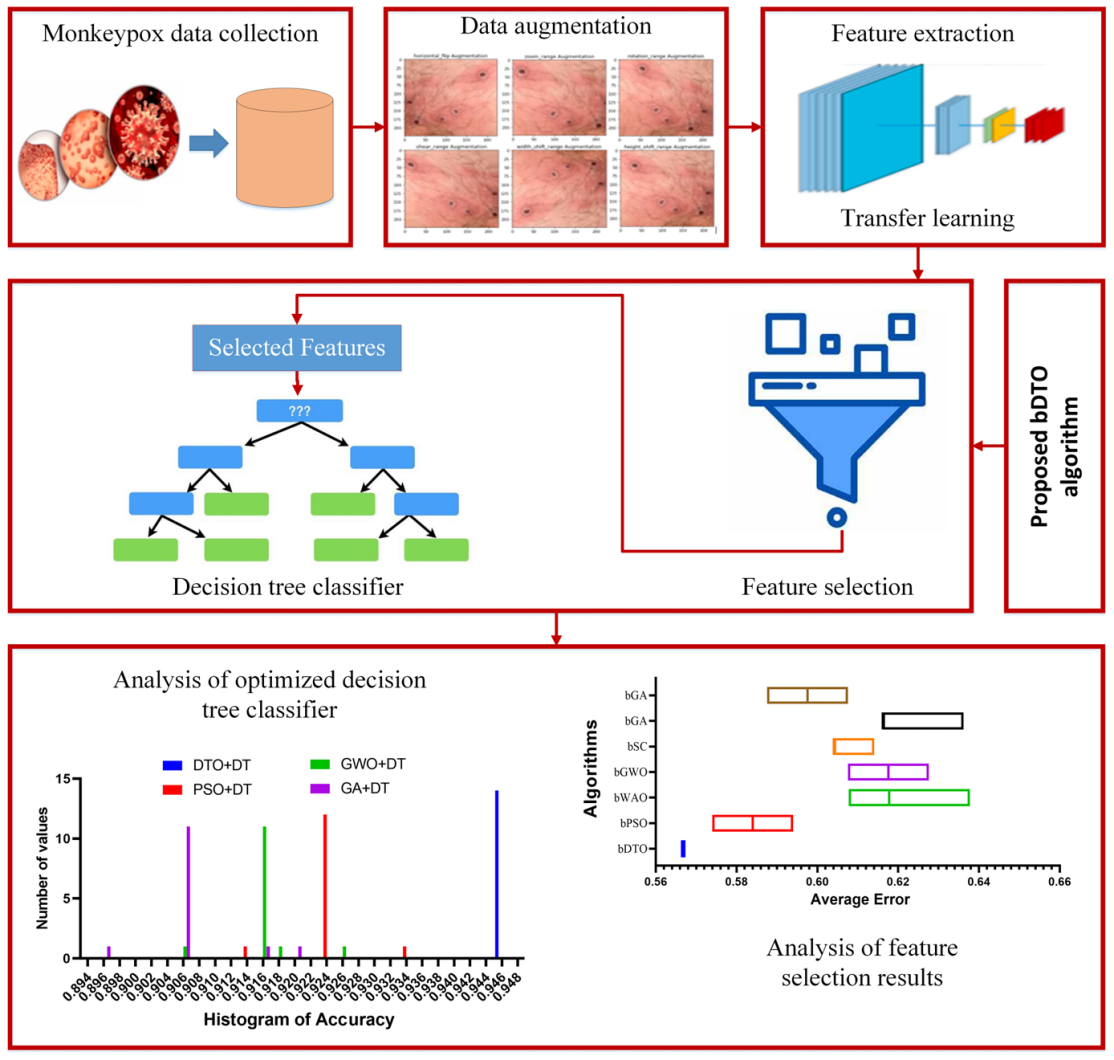
The general architecture of the proposed methodology.

The proposed procedure has six primary steps: collecting the proper dataset, data augmentation, feature extraction, optimized feature selection, model training, and optimization, testing, and statistical evaluation. The gathering of monkeypox images is realized by adopting a dataset freely available on Kaggle. Data augmentation techniques are utilized to generate new images due to a limited number of raw data in the adopted dataset. Four widely used deep learning models, namely AlexNet [48], VGG19 [49], ResNet50 [50], and GoogleNet [51], are trained, tested, and compared to select the best model that achieves the best model accuracy in detecting monkeypox cases. To train the chosen models, the preprocessed images are fed into the model, and its parameters are tuned throughout training to improve accuracy. The model that achieves the best performance is then used to extract the features from the input images. The extracted features are then processed using the proposed feature selection algorithm to select the best set of features. This best set of features is used to train an optimized decision tree classifier to decide the case of the input image. At this point, the model is put through its paces, measuring its efficacy with statistics such as accuracy, precision, recall, and F1-score. Figure 1 shows the steps and processes that comprise the proposed methodology.

### 3.1. Data Collection

The Monkeypox Skin Image Dataset, available on Kaggle [52,53], is adopted for the experiments conducted in this paper. This dataset is a valuable resource for researchers and medical professionals studying the monkeypox virus and its impact on human health. This dataset comprises a collection of high-resolution images depicting various stages and manifestations of monkeypox infection on human skin. Monkeypox is a rare viral disease that causes a pox-like rash and is similar to smallpox in its symptoms. The dataset provides a comprehensive visual representation of the disease, including images of skin lesions, rashes, and other related dermatological conditions. With a wide range of images capturing different aspects of monkeypox, this dataset offers researchers an opportunity to explore the progression of the disease and identify distinguishing features that can aid in accurate diagnosis. Moreover, the dataset can serve as a valuable resource for developing and validating computer vision algorithms or machine learning models aimed at automating the detection and classification of monkeypox-related skin conditions. By leveraging this dataset, researchers can gain insights into the visual characteristics of monkeypox and potentially contribute to the development of improved diagnostic tools and treatment strategies. The availability of such a comprehensive and specialized dataset promotes collaboration and facilitates advancements in the field of dermatology and infectious diseases. This dataset consists of 770 images divided into 60% for training, 20% for validation, and 20% for testing.

### 3.2. Data Preprocessing

Preprocessing is vital to image analysis because it raises the bar for data quality and uniformity. The paper’s preliminary processing procedures included image scaling, image normalization, and data enhancement.

#### 3.2.1. Image Resizing and Image Normalization

This section presents the preprocessing methods used in earlier trials, such as image scaling, which allows researchers to adjust the input images to suit the DL better. This method mimics a real-time experiment by performing the resizing process using a fully convolutional layer as part of the network, even though it is implemented before the images are fed into the models. The images in this study were scaled down to 224 × 224 pixels so that the model could more easily process the data because of the uniformity in the size of the images. Differences in lighting and contrast were smoothed out by applying a normalization filter to the images. The image’s pixel values were then transformed from their original range of 0–255 to a more manageable 0–1.

#### 3.2.2. Data Augmentation

**Figure 2:**
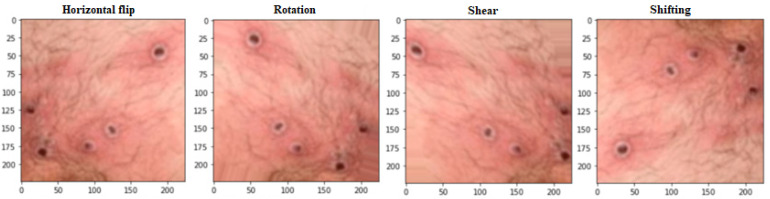
The implemented operations of data augmentation.

Figure 2 demonstrates how data augmentation techniques were applied to the images to increase the dataset size and prevent overfitting. The model’s accuracy and reliability were improved due to this expansion of the data set. Many data augmentation techniques have been proposed [54,55] to address these two obstacles. Regular data augmentation techniques can increase the dataset’s size progressively. Some examples of these techniques are flipping, rotation (0–360 degrees), shearing, and shifting. A larger and more varied dataset can be created for training the deep learning model with the help of these methods, which allow for developing new images with minor alterations to the original images. By adding more information to the dataset, the proposed data augmentation methods hope to boost the model’s generalization ability. The model is better equipped to learn the fundamental aspects of the images when these methods are used since it is exposed to a broader variety of variants. The use of these methods to expand the dataset improved the accuracy and reliability of classifying monkeypox images. The size of the dataset after the process of data augmentation is 2500 images.

### 3.3. Feature Extraction

**Figure 3:**
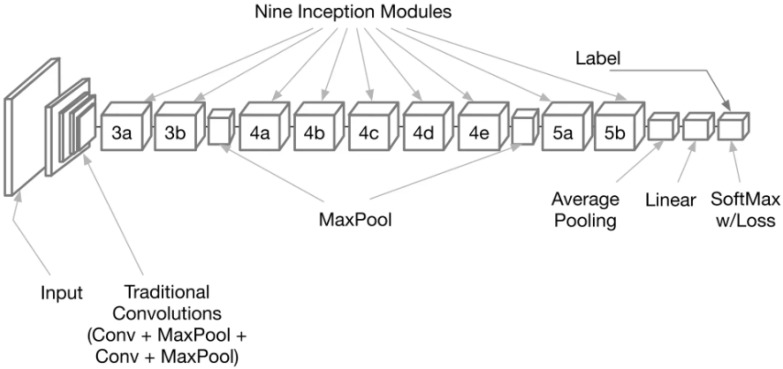
The general architecture of the GoogLeNet deep network. In this figure, the numbers 3,4, and 5 refer to the number of layers in this composite node.

With deep learning frameworks like GoogLeNet, the feature extraction process can be broken down into many stages. Obtaining a dataset of monkeypox images and preparing them to eliminate noise and artifacts that could compromise feature extraction is the first stage in data preparation. The images are first separated into three sets to train and evaluate models: training, validation, and testing. Step two entails deciding on what kind of model to use and how to structure the system. The model’s architecture is then created by choosing parameters such as the number of layers, the size of the filters in the convolutional layers, the type of pooling layers, and the number of neurons in the fully connected layers. After the data is prepared, the GoogLeNet model, shown in Figure 3, is fed the data, and the network is trained by backpropagation to alter the weights and biases of the neurons to minimize the loss function [31,56,57,58]. Here, the advantage of the power and efficiency of deep learning frameworks like GoogLeNet to extract features from images with pinpoint precision is exploited.

The network must be trained to recognize certain patterns and features to classify monkeypox images. Training entails tinkering with the model’s hyperparameters—like learning rate and batch size—to obtain the best results. At this point, the trained network’s intermediate layers are probed for their learned features, which leads us to step four: feature extraction. Important patterns and properties of the monkeypox virus that can be utilized for categorization can be identified using these features. In addition to their usage in visualization and analysis, the retrieved features can also be used in other ways. Step five, model evaluation, involves determining how well the model predicts data from the testing set. Predicted labels are compared to the actual labels of images in a testing set to determine how well they match up. If the model’s performance is adequate, it can be used to categorize new monkeypox images. In conclusion, employing deep learning frameworks such as GoogLeNet for feature extraction necessitates a multi-stage process that begins with data preparation and continues through model selection and architecture design, model training, feature extraction, and model evaluation. New monkeypox images can be seen, analyzed, and classified using the acquired features.

### 3.4. Feature Selection

The dipper bird’s ability to dive deep into the water in pursuit of food inspired the development of a binary optimization technique known as binary dipper throated optimization (bDTO) [59]. The technique can be used in feature selection for machine learning tasks where it can pick out the most important characteristics from a pool of data retrieved using a deep neural network like that of Google. What follows is a brief overview of how bDTO is used to select the best set of features:

- The input features’ continuous values must be encoded as binary strings using the binary encoding method. The threshold value determines which numbers are considered significant enough to be assigned binary values of 1 and 0, respectively. Its binary encoding simplifies the optimization problem by reducing the search space to which it must be applied.
- During the second phase, “initialization”, bDTO generates a population of “dipper birds”, where each bird represents a possible feature subset. There is a random seed used to start the population.
- The fitness function evaluates the subset of features for each bird to determine how good they are. The accuracy of a classifier trained with the bird-representative subset of features could serve as the fitness function in the case of monkeypox image classification.
- Fourth, bDTO uses the fitness function to guide the birds’ relocation to the most beneficial subsets of characteristics. The size of the steps, the strength of the attraction, and the strength of the repulsion govern the motion. The step size determines how far a bird may fly throughout each repetition. The birds are drawn to the optimal subsets of features by the attraction parameter and are kept from settling into a rut by the repulsion parameter.
- Fifth, bDTO performs crossover and mutation procedures on the birds during the flight, resulting in new generations. Unlike mutation, which changes the values of random bits in a binary string, crossover requires joining the binary strings of two birds to generate a new one. The offspring take on the fitness-based competition depending on the feature subset they inherited from their parents.
- Sixth, a selection is made once the children have been produced using bDTO employing the fitness values of each individual to determine which subsets of traits are most advantageous. For the next iteration of the algorithm, a new population is generated using the features that were narrowed down during the previous one.
- When a stopping criterion is reached, the migration, hybridization, mutation, and selection process are terminated. The halting criterion may be an absolute maximum runtime, a minimal increase in fitness value, or some combination of the two.

**Figure 4:**
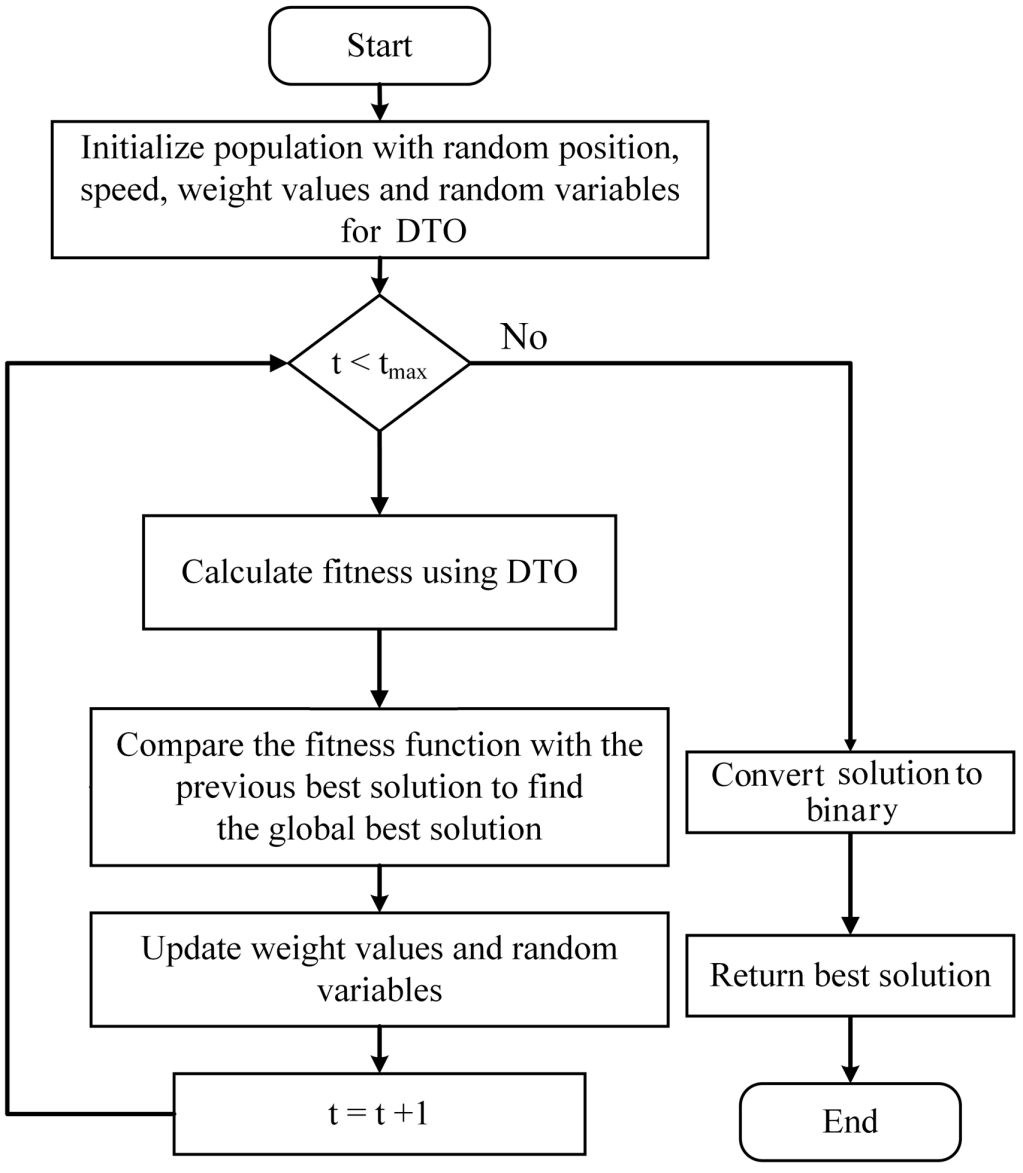
The feature selection process using the proposed bDTO algorithm.

In summary, bDTO takes the input features’ continuous values and turns them into binary, then generates a flock of dipper birds to represent the features’ possible subsets. The fitness function measures the quality of each bird’s feature subset. The optimal feature subset is chosen by comparing fitness values, and this process is continued until a stopping threshold is reached. The process of the proposed bDTO algorithm is shown in Figure 4.

## 4. Evaluation Results

In this section, the findings of an analysis of the classification performance of the suggested method are presented and discussed. The deep learning models used in this research were trained, tested, and validated using the adopted dataset.

### 4.1. Evaluation Criteria

Using the confusion matrix, it is possible to compute measures like accuracy, precision, recall, and F1-score by comparing the predicted labels to the genuine ones. True positives (TPs), true negatives (TNs), false positives (FPs), and false negatives (FNs) are the four categories that make up the confusion matrix (FNs). When the actual class is monkeypox and the predicted class is monkeypox as well [60], the case is correctly predicted and denoted with a TP. Those circumstances where the actual and expected classes are not monkeypox are referred to as “true negatives” (TNs). False positives (FP) are cases where the expected class is monkeypox but the actual class is not. False negatives (FNs) represent cases where monkeypox is the real class but the projected class is different. The best-performing model was chosen as the gold standard for identifying and categorizing cases of monkeypox.

Accuracy is how often a model produces accurate predictions in relation to how often it produces incorrect predictions. This can be written down mathematically in the form below.

(1) $$Accuracy=\frac{\left(TN+TP\right)}{\left(TN+FN+FP+TP\right)}.$$

Precision (*p*-Value) is defined as the percentage of predicted positive cases that materialize as opposed to the total number of positive cases Precision, as it pertains to this study, is defined as the proportion of true positive predictions of monkeypox to the overall predictions of monkeypox. The following is a mathematical representation of this criterion. In addition, the N-Value is used to measure the opposite percentage. The measures of *p*-Value and N-Value are performed using the following equations.

(2) $$p-Value=\frac{TP}{\left(FP+TP\right)},$$

(3) $$N-Value=\frac{TN}{\left(TN+FN\right)}.$$

The term “Sensitivity” refers to the rate at which a model accurately forecasts future instances of monkeypox. The accuracy of a test depends on how many true positives it predicts compared to how many there are. The following is the mathematical representation of this.

(4) $$Sensitivity=\frac{TP}{\left(FN+TP\right)}.$$

When the costs of false negatives and false positives vary, the F1-Score, a weighted average of precision and recall, is a more appropriate indicator than accuracy. This is a proof that can be demonstrated mathematically.

(5) $$F1-Score=\frac{\left(2\times Precision\times Recall\right)}{\left(Precision+Recall\right)}.$$

The F1-score is preferable to accuracy when the cost of false negatives and false positives is equivalent. On the other hand, if the prices for accuracy and recall vary, it is best to weigh both. In addition, the specificity calculation is performed in terms of the following equation.

(6) $$Specificity=\frac{TN}{\left(TP+FP\right)}.$$

### 4.2. Feature Extraction Results

Accuracy, sensitivity, specificity, *p*-Value, N-Value, and F1-score are just some of the metrics used in determining the quality of the extracted features. Correctly classified instances as a percentage of all instances is the definition of accuracy. It is often employed as a measurement of classification model efficacy. The percentage of real positive cases that are accurately classified as positive is the measure of sensitivity. The percentage of false-negative cases that were accurately detected is what specificity is based on. If the null hypothesis is correct, then the *p*-Value is the likelihood that a result as extreme as the one obtained would be seen. The N-Value indicates how many features were taken into account. At last, the F1-score is a popular metric for assessing classification model efficacy, calculated as the weighted average of precision and recall. In most cases, a high sensitivity score suggests that the features can accurately detect positive examples, while a high accuracy score indicates that the features are relevant and valuable for the classification task. Their specificity measures the characteristics’ ability to detect negative instances accurately. When the *p*-Value is low, the results are statistically significant, and the extracted features were not generated by coincidence. When the N-Value is high, it means that many features have been retrieved, which may or may not be useful, depending on the context. If a classification task is being performed, an F1-score that is high is desirable since it suggests a good balance between precision and recall. These metrics allow us to judge the efficacy of features retrieved by deep learning frameworks like GoogleNet. It is possible to infer that the feature extraction procedure has successfully identified the most relevant features for the classification task at hand if the extracted features have a high accuracy, sensitivity, specificity, and F1-score, and a low *p*-Value as presented in Table 2. In this table, the results using the feature extracted based on the GoogleNet are better than those of the other deep networks. Therefore, this network is adopted for further steps in the proposed methodology.

### 4.3. Feature Selection Results

Once the feature extraction process is completed, feature selection techniques are used to narrow down the collected features further. Best fitness, worst fitness, average error, average fitness, average select size, and standard deviation fitness are only some metrics used to assess the performance of the features we have chosen. The term “best fitness” describes the highest fitness value that the optimized feature subset has attained. This statistic matters because it shows the best possible classification performance using the chosen feature subset. On the other hand, the worst fitness is the lowest fitness value that the chosen feature subset obtained during optimization. The lowest possible classification performance that can be achieved with the chosen feature subset is indicated by this statistic, making it crucially essential. When discussing classification, “average error” refers to the typical rate of mistakes made by the features chosen for inclusion in the subset. This statistic matters because it gives a rough idea of how well the classification model works with the chosen subset of features. During optimization, a subset of features is chosen, and its fitness is measured on average to determine its overall fitness. This measure is useful since it gives an idea of how well the chosen feature subset can separate classes. “Average select size” means the typical number of features the algorithm chooses for optimization. An estimate of the classification model’s complexity and the number of features required for acceptable performance may be gleaned from this statistic, making it a useful quality indicator. Lastly, the fitness standard deviation quantifies the range of possible outcomes based on the subsets of features that were chosen for optimization. This statistic matters because it provides insight into the reliability of the optimization procedure and the strength of the prioritized set of features. The quality, complexity, stability, and robustness of the selected feature subset and the possession of valuable insights into the performance of the classification model can be measured by using best fitness, worst fitness, average error, average fitness, average select size, and standard deviation fitness to evaluate the results of feature selection. Table 3 presents the evaluation criteria results based on the proposed feature selection method with a comparison to other methods, namely binary particle swarm optimization (bPSO), binary whale optimization algorithm (bWOA), binary gray wolf optimization (bGWO), binary sine cosine (bSC), binary genetic algorithm (bGA), and binary firefly algorithm (bFA). In this table, it can be noted that the results of the proposed feature selection method outperform those of the other feature selection methods in the literature. These results prove the superiority and effectiveness of the proposed method in selecting the significant set of features necessary for classifying monkeypox cases.

**Figure 5:**
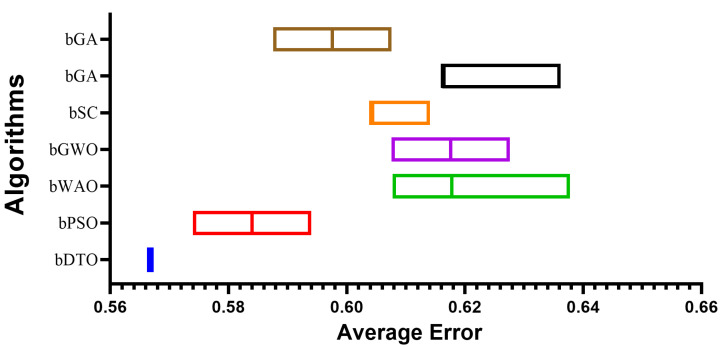
The average error using the proposed feature selection method in comparison to other methods.

To provide a more clear picture of the performance of the proposed feature selection method, Figure 5 depicts the average effort when using the proposed feature selection methods and the other methods. In this figure, the proposed feature selection method achieves the lowest average error, indicating the best performance among the other methods included in the experiment.

In terms of statistical analysis, the analysis of variance (ANOVA) test has been performed, and the results are presented in Table 4. The results in this table show that the suggested bDTO feature selection algorithm differs significantly from the other feature selection methods, as shown by the ANOVA findings presented. The Treatment (between columns) row demonstrates this with a massive F-Value of 205.0 and a tiny *p*-Value of less than 0.0001. Given that the residual variance is only 0.001833, this shows that there is not much variation between the different feature selection methods. This suggests that the feature selection technique effect is primarily responsible for the observed variation in the outcome measure. Variation in the outcome measure was calculated across all groups and was found to be a total of 0.03111 in the sum of squares. While the ANOVA findings show that the bDTO feature selection algorithm outperforms the other feature selection methods on the outcome measure, it is vital to remember that this is merely an indication. The findings do not indicate how big the effect is or in which direction it is going, and they do not indicate how bDTO differs from the other feature selection approaches. Extra post hoc evaluations could be required to determine which feature selection strategies are distinct from bDTO. Based on the ANOVA findings, it may be worthwhile to further investigate the efficacy of the proposed bDTO feature selection algorithm in practical settings, as it shows promise and differs significantly from previous feature selection approaches. It should be noted, however, that more testing and validation are required to assess the performance of the bDTO feature selection method in a wider range of contexts.

To check whether two samples are drawn from the same population, a non-parametric technique called the Wilcoxon signed-rank test can be performed. The results of this test are presented in Table 5. In this table, the evaluation served to contrast the effectiveness of the suggested bDTO feature selection algorithm with that of competing approaches. According to the findings, bDTO outperformed the other approaches, as its real median was more significant than the median of all the other methods. The total number of signed ranks for all approaches was 78, which indicates a large spread in results for the various feature selection strategies. Since the *p*-Value is less than 0.05, it is highly unlikely that these results were obtained by chance. Since the sample size was small enough, the exact test was performed instead of an approximate one. bDTO’s superior performance was demonstrated by the smallest disparity between the theoretical and observed medians across all methods. These findings collectively point to bDTO as a potential feature selection technique that outperforms the majority of currently utilized approaches.

**Figure 6:**
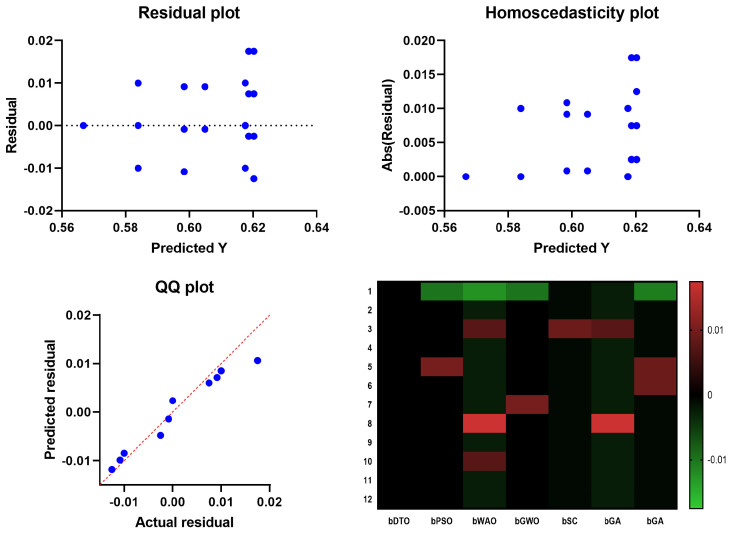
The analysis of the results achieved by the ANOVA test.

In Figure 6, the Quartile–Quartile (QQ) plot is a graphical method for determining the normality of a data set. When contrasting the efficacy of various feature selection techniques, a QQ plot can be used to visualize how each technique’s residuals (the gaps between the anticipated and actual values) compare. A straight line appears in a QQ plot if the residuals follow a normal distribution. One indicator of a method’s ability to predict an outcome variable is whether or not its residuals depart from the straight line more than competing methods. In regression analysis, homoscedasticity refers to the hypothesis that residuals are normally distributed throughout all levels of the predictor variables. Checking for homoscedasticity involves graphing the residuals against the anticipated values for each feature selection method. The assumption of homoscedasticity, in which the residuals have the same variance regardless of which predictor variables are used, is met if the residuals are normally distributed around a horizontal line. The assumption of equal variance across predictor variables is violated if the residuals exhibit a discernible pattern or grow or drop consistently as the predicted values increase, which indicates that the residuals are heteroscedastic. The residuals are plotted vertically against the projected values in a scatter plot called a residual plot. Residual plots are helpful for visually evaluating the fit of the models in the context of contrasting feature selection strategies. The ideal distribution of residuals would have no discernible trends and be uniformly distributed along a horizontal axis. There may be a problem with the model’s ability to capture the relationship between the predictors and the outcome variable if the residual plot has a discernible pattern, such as a curve or a systematic increase or drop. The relationship between the predictor variables and the outcome can be shown in a heatmap plot. When contrasting feature selection strategies, a heatmap plot can reveal which predictors are highly and weakly linked with the outcome variable. Selecting predictors with a high correlation to the outcome variable regularly indicates a successful feature selection approach. Compared to other feature selection approaches, the suggested bDTO method’s performance may be evaluated using these charts. If the bDTO technique’s residuals show a large departure from the straight line in the QQ plot, this may indicate that the method is underperforming compared to alternatives. If the bDTO method’s residual plot has a discernible pattern, it indicates that the model is not successfully representing the connection between the predictors and the outcome variable. If the heatmap plot reveals that the bDTO technique reliably chooses predictors with a high correlation to the outcome variable, this indicates the method’s success.

### 4.4. Classification Results

The next step is to use a classification algorithm on the extracted and refined set of features. Specifically, a decision tree classifier is utilized to sort the monkeypox images. A tree-like model of decisions and their outcomes was constructed using the decision tree classifier. The DTO algorithm, a population-based metaheuristic optimization approach, was utilized to achieve optimal performance in the decision tree classifier. The optimization method aims to discover the optimum decision tree model by exploring all possible decision trees to improve classification accuracy. Accuracy, sensitivity, specificity, precision, recall, and F1-score are just a few measures that may be used to assess the optimized decision tree classifier’s efficacy. As evaluated by these metrics, high classification performance is possible with the optimized decision tree classifier if the features chosen are insightful and can accurately distinguish between the various monkeypox image classes. Table 6 presents the classification results before and after feature selection. It is obvious from this table that the classification results using the proposed feature selection are better than those of the classification with prior feature selection.

**Figure 7:**
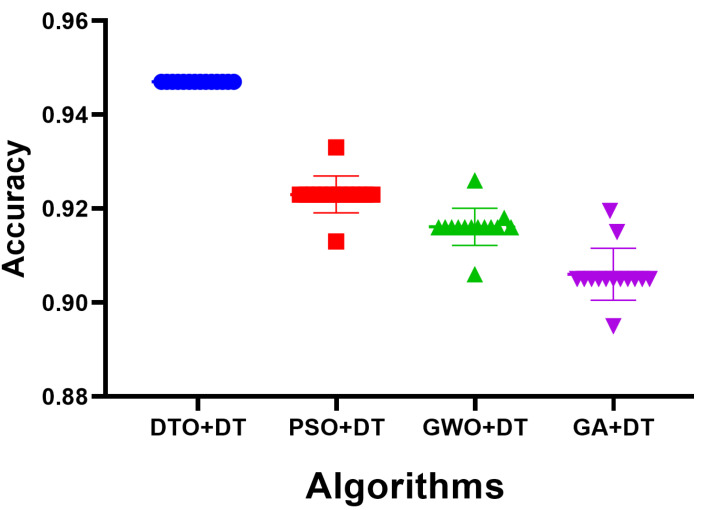
The accuracy achieved by the decision tree classifier when optimized using the proposed DTO algorithm in comparison to the other optimization algorithms.

**Figure 8:**
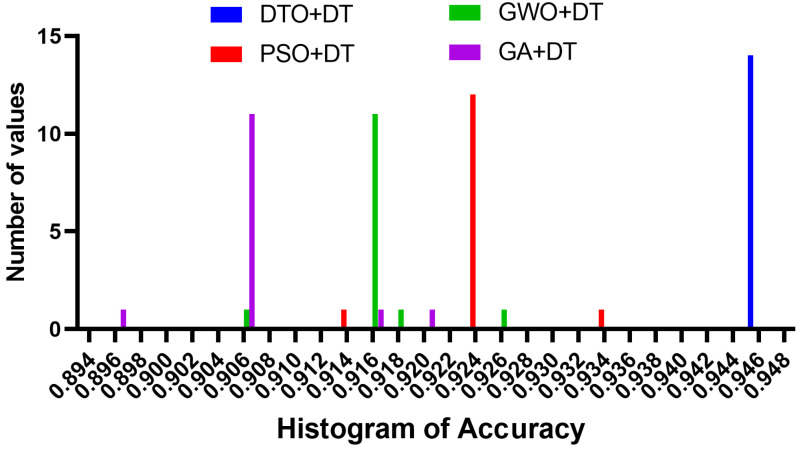
The histogram of the accuracy achieved by the proposed approach in comparison to the other algorithms.

As the results using the proposed feature selection are promising, the selected features are used as input for the optimized decision tree classifier. When fed with the selected feature, the optimized classifier’s results are shown in Figure 7. The achieved accuracy is measured and shown in the plot in this figure. The accuracy of the proposed approach is 94.35%, which is better than the accuracy achieved when the decision tree is optimized using different optimization algorithms. On the other hand, to show the stability of the resulting accuracy, the histogram shown in Figure 8 proves the stability of the achieved results as most of the test cases have been classified with high accuracy. However, the other approaches have varying classification accuracy depending on the test cases. These results confirm the stability of the proposed methodology.

### 4.5. Statistical Analysis

**Figure 9:**
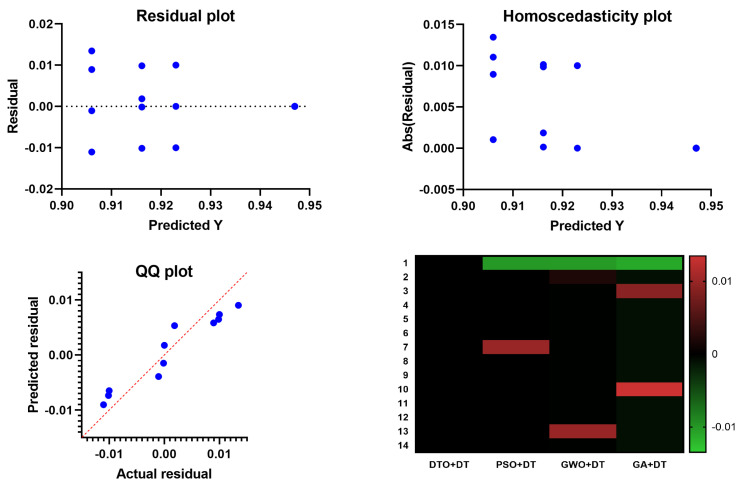
The analysis of the results achieved by the ANOVA test.

An additional experiment was conducted to study the statistical properties of the proposed methodology. This study focused on the statistical difference between the proposed methodology and other methodologies used in optimizing the decision tree classifier. The statistical analysis was performed in terms of the statistical analysis in Table 7, the analysis of variance (ANOVA) test in Table 8, and the results are presented and the Wilcoxon signed rank test in Table 9. These results prove the statistical difference between the proposed classification approach when compared to the other approaches. In addition, the plots in Figure 9 show the performance regarding the residual, homoscedasticity, QQ, and heat map. These results confirm the significance of the proposed methodology and the statistical difference as the *p*-Value is less than 0.005.

## 5. Discussion

Positive results in terms of accuracy, precision, recall, and F1-score were found when the models were evaluated using the modified dataset in comparison to previous work in the field. The suggested model is compared to AlexNet, ResNet50, and GoogLeNet, all of which have already been pre-trained as deep learning models. Our proposed model was shown to have the highest accuracy, precision, recall, and F1-score of all models tested. Our proposed model and other models performed well on this dataset, with the proposed optimized decision tree classifier obtaining the highest accuracy score of 94.35%. As can be seen from the evaluation results, the suggested model performs well compared to other deep learning models on the provided dataset and is also competitive in terms of fitness. To perfect the proposed model, both unaltered and enriched training data are used in its development. When fewer instances are available, this method can improve performance. As a result of applying augmentation to the whole dataset, there is a significant disparity between the validation and testing data. The vast majority of instances involve something other than monkeypox. However, when augmentation was given only to the training data, both the validation and testing data became skewed; nevertheless, in most instances, the skewness was due to the monkeypox. The models performed effectively, with only a single case being misclassified despite the asymmetry in the data.

Our primary contribution is describing a cutting-edge machine learning approach to monkeypox detection that can vastly enhance diagnostic throughput and precision. This may help speed up the process of identifying and treating monkeypox. Due to its impressive performance on both the training and test sets, the proposed model has great promise as a useful resource for the rapid and precise diagnosis of monkeypox in clinical settings. Medical professionals may be able to improve patient outcomes and decrease healthcare expenses if this study’s findings are implemented. The suggested model performs more accurately and efficiently compared to several other models. The suggested model can be used in real time as an assessment tool, and it might be applied on smartphones for the real-time identification and forecasting of monkeypox cases. Due to its impressive performance on both the training and test sets for monkeypox classification, the proposed model has the potential to be a valuable tool for efficient clinical diagnosis. Additionally, studies involving automated monkeypox detection utilizing deep learning and transfer learning techniques may lead to new diagnostic tools and methods for other infectious diseases. This would be a significant step forward in healthcare and disease diagnosis.

## 6. Conclusions and Future Work

The primary purpose of this research was to improve feature selection and classification methods for spotting skin lesions indicative of monkeypox in the event of a pandemic by employing metaheuristic optimization. Deep learning and a transfer learning strategy are employed to obtain the proper set of features. To accomplish this task, a deep learning framework known as the GoogLeNet network is employed. DTO is used for feature selection and is implemented in a binary form. Once a set of features is chosen, it can be labeled using a decision tree classifier. A continuous implementation of the DTO method is used to optimize the decision tree classifier, leading to better accuracy in the classification process. Accuracy, sensitivity, specificity, *p*-Value, N-Value, and F1-score were used to evaluate and compare the different approaches. The F1-score was 0.92, the sensitivity was 0.95, the specificity was 0.61, the *p*-Value was 0.89, and the N-Value was 0.79, all from using a decision tree classifier and selecting features. After fine-tuning the decision tree classifier’s parameters, the proposed technique achieves an overall accuracy of 94.35%. In addition, the results have been subjected to ANOVA and the Wilcoxon signed-rank test to examine the statistical differentiation between the suggested methodology and the alternatives. This contrast proved the superiority of the proposed method for identifying monkeypox cases. Future perspectives of this work include addressing cutting-edge machine learning models, such as transformers and state-of-the-art models, along with optimization algorithms to boost the overall performance of the proposed methodology.

## Figures and Tables

**Table 1:** Review of related works.

Paper	Model	Method	Purpose
[41]	VGG-16, ResNet50, and InceptionV3 models	Monkeypox skin lesion detection using deep learning models	Monkeypox skin lesion detection
[42]	Xception, DenseNet	Detection of monkeypox virus by transfer learning methods	Monkeypox virus detection
[43]	They propose and evaluate the VGG16 model with D curve	Image data collection and implementation of a deep-learning-based model in detecting monkeypox disease	Detecting monkeypox disease
[44]	GoogleNet, EfficientNetb0, NasnetMobile, ShuffleNet, MobileNetv2 models	Human monkeypox classification from skin lesion images with deep pre-trained network	Human monkeypox classification from skin lesion images
[45]	ResNet50	Convolutional neural networks with transfer learning to diagnose Lyme disease from skin lesion	Lyme disease from skin lesion images
[46]	Resnet50	Automated detection of erythema migrans and other confounding skin lesions via deep learning	Automated detection of erythema migrans
[47]	EfficientNetV2s, MobileNetV3, VGG19, ResNet50, DenseNet	Monkeypox detection using CNN with transfer learning	Monkeypox detection
This work	GoogleNet and Metaheuristic Optimization	Monkeypox detection using CNN with transfer learning	Monkeypox detection

**Table 2:** Evaluating the features extracted using the GoogLeNet deep network.

	Accuracy	Sensitivity	Specificity	*p*-Value	N-Value	F1-Score
AlexNet	0.80	0.91	0.56	0.81	0.75	0.86
VGG19	0.80	0.91	0.56	0.82	0.75	0.86
ResNet50	0.81	0.92	0.56	0.83	0.75	0.87
GoogLeNet	0.82	0.93	0.56	0.84	0.75	0.88

**Table 3:** Assessment of the selected set of features using the proposed optimization algorithm compared to other optimization algorithms.

	bDTO	bPSO	bWOA	bGWO	bSC	bGA	bGA
Best Fitness	0.53	0.57	0.62	0.62	0.63	0.62	0.56
Worst Fitness	0.63	0.63	0.69	0.69	0.71	0.71	0.68
Average Error	0.57	0.58	0.62	0.62	0.60	0.62	0.60
Average Fitness	0.63	0.65	0.64	0.65	0.65	0.70	0.66
Average Select size	0.52	0.72	0.72	0.88	0.64	0.75	0.66
Standard Deviation Fitness	0.45	0.46	0.46	0.46	0.46	0.49	0.46

**Table 4:** The results of the analysis of variance test.

ANOVA Table	SS	DF	MS	F (DFn, DFd)	*p*-Value
Treatment (between columns)	0.02928	6	0.00488	F (6, 77) = 205.0	*p* < 0.0001
Residual (within columns)	0.001833	77	0.00002381		
Total	0.03111	83			

**Table 5:** The results of the Wilcoxon signed-rank test.

	bDTO	bPSO	bWOA	bGWO	bSC	bGA	bGA
Theoretical median	0	0	0	0	0	0	0
Actual median	0.5668	0.584	0.6178	0.6176	0.6041	0.6162	0.5976
Number of values	12	12	12	12	12	12	12
Sum of signed ranks (W)	78	78	78	78	78	78	78
Sum of +ve ranks	78	78	78	78	78	78	78
Sum of −ve ranks	0	0	0	0	0	0	0
*p*-value	0.0005	0.0005	0.0005	0.0005	0.0005	0.0005	0.0005
Exact or estimate?	Exact	Exact	Exact	Exact	Exact	Exact	Exact
Discrepancy	0.5668	0.584	0.6178	0.6176	0.6041	0.6162	0.5976

**Table 6:** The classification results with and without implementing the proposed feature selection method.

	Accuracy	Sensitivity	Specificity	*p*-Value	N-Value	F1-Score
Before FS	0.85	0.94	0.61	0.87	0.79	0.90
After FS	0.87	0.95	0.61	0.89	0.79	0.92

**Table 7:** The results of the statistical analysis.

	DTO + DT	PSO + DT	GWO + DT	GA + DT
Number of values	14	14	14	14
Minimum	0.947	0.913	0.906	0.895
25% Percentile	0.947	0.923	0.916	0.905
Median	0.947	0.923	0.916	0.905
75% Percentile	0.947	0.923	0.916	0.905
Maximum	0.947	0.933	0.926	0.9195
Range	0	0.02	0.02	0.0245
10% Percentile	0.947	0.918	0.911	0.9
90% Percentile	0.947	0.928	0.922	0.9173
95% CI of median				
Actual confidence level	98.71%	98.71%	98.71%	98.71%
Lower confidence limit	0.947	0.923	0.916	0.905
Upper confidence limit	0.947	0.923	0.916	0.905
Mean	0.947	0.923	0.9161	0.906
Std. Deviation	0	0.003922	0.003959	0.005514
Std. Error of Mean	0	0.001048	0.001058	0.001474

**Table 8:** The results of the analysis of variance test.

	SS	DF	MS	F (DFn, DFd)	*p* Value
Treatment	0.01275	3	0.00425	F (3, 52) = 276.6	*p* < 0.0001
Residual	0.0007989	52	1.54 × $10^{-5}$		
Total	0.01355	55			

**Table 9:** Wilcoxon test results.

	DTO + DT	PSO + DT	GWO + DT	GA + DT
Theoretical median	0	0	0	0
Actual median	0.947	0.923	0.916	0.905
Number of values	14	14	14	14
Wilcoxon Signed-Rank Test				
Sum of signed ranks (W)	105	105	105	105
Sum of positive ranks	105	105	105	105
Sum of negative ranks	0	0	0	0
P value (two-tailed)	0.0001	0.0001	0.0001	0.0001
Exact or estimate?	Exact	Exact	Exact	Exact
Significant (alpha = 0.05)?	Yes	Yes	Yes	Yes
How big is the discrepancy?				
Discrepancy	0.947	0.923	0.916	0.905
